# Special Issue: Host–Fungus Interactions

**DOI:** 10.3390/jof4010007

**Published:** 2018-01-02

**Authors:** Adilia Warris

**Affiliations:** Aberdeen Fungal Group, MRC Centre for Medical Mycology, University of Aberdeen, Foresterhill, Aberdeen AB25 2ZD, UK; a.warris@abdn.ac.uk

Dear Colleagues,

The clinical presentation of fungal disease is strongly determined by the underlying immune defect present [1]. In particular, over the last two decades, the increased recognition of monogenic disorders (e.g., primary immunodeficiencies) have provided us with valuable insights into human immunity to fungal infections. The discovery of those so-called “experiments of nature” have been crucial in the elucidation of underlying molecular mechanisms with the ultimate aim to develop new treatment modalities. 

Genetic polymorphisms resulting in defects in *Candida* recognition, Th17 differentiation and proliferation or IL-17R signalling can increase susceptibility to chronic mucocutaneous candidiasis, and lend evidence for the importance of the Th17/IL-17axis in antifungal immunity. Mengesha and Conti describe in this Special Issue the current insights in the molecular pathways involved in IL-17 mediated antifungal immunity [2]. They also refer to the potential risks of fungal infectious complications associated with the use of anti-IL-17 antibodies in the treatment of auto-immune diseases. The current molecular insights, although most likely far from complete, have already resulted in new therapeutic leads and are currently being investigated [3]. 

An excellent review written by Heung provides us with a valuable update on the progress made in deciphering the immune responses and molecular pathways to resist cryptococcal infections [4]. Next to adaptive immune cells, several innate immune cell types appear to have effector functions to combat cryptococcal disease. In parallel, identification of non-HIV patients with specific immune defects rendering them susceptible to cryptococcosis have recently been described [5].

Meya et al. demonstrate the results of their study in this Special Issue that monocyte subset phenotype and cytokine responses prior to antiretroviral therapy may be predictive of cryptococcal meningitis immune reconstitution inflammatory syndrome (CM-IRIS) [6]. Although the study population was small, 17 HIV patients of which 11 developed CM-IRIS, the absence of non-classical monocytes at CM diagnosis and an increased IFNγ induced IL-6 and TNFα expression by monocytes differentiated those patients developing CM-IRIS. Those new results in combination with previous clinical and experimental data suggest that immunomodulatory therapy to neutralize and/or suppress IL-6 and/or TNFα may be an attractive approach to prevent or improve the outcome of CM-IRIS. 

Chronic pulmonary aspergillosis is observed in patients with a wide variety of underlying lung diseases, although no specific immune defects associated with the development of CPA have been identified. Obviously, the damaged lung tissue by the primary lung disease may facilitate Aspergillus conidia to persist and germinate into hyphal airway invasive structures, leading to pulmonary aspergillosis. Since only a subset of patients with a defined underlying lung disease develops CPA, the following question arises: Do those patients suffer from additional defects in their immune system? Bongomin et al. have made a first attempt to look into this by analysing immunophenotyping of lymphocyte subsets in peripheral blood samples of 144 patients with CPA [7]. Interestingly, 12.5% of the patients had a subnormal NK-cell (CD56) count and the role of NK-cells in antifungal immunity has been nicely reviewed by Schmidt et al. in this Special Issue [8]. They refer to two clinical observational studies in post-haematopoietic stem cell and post-solid organ transplantation patients demonstrating an association with poor function and/or low numbers of NK-cells and the development of invasive aspergillosis. In both studies, as well as in the study presented by Bongomin in this Special Issue, it nevertheless remains unclear if the low number of NK-cells caused *Aspergillus* disease to develop. The challenge to be aimed for is to perform longitudinal clinical studies in well-defined patient groups to demonstrate a causative relationship between abnormal lymphocyte subsets and the development of CPA. 

The increased insight in the characteristics of polysaccharides in the fungal cell wall, its recognition by specific pathogen receptors, and the downstream signalling resulting in either up- or down-regulation of inflammatory pathways is the subject of the review by Snarr et al. in this Special Issue [9]. In addition, the authors refer to the promising potential of this new knowledge with respect to the development of antifungals with a new mode of action and the use of immunosuppressive fungal polysaccharides in the treatment of inflammatory disorders as rheumatoid arthritis. On the other hand, Hernandez-Chavez et al. present us with a clearly written overview of how fungal cells have evolved several strategies to avoid recognition by immune cells, including the modulation of the amount of certain cell wall components (e.g., polysaccharides) or their accessibility on the fungal cell surface [10]. 

The ability of *C. albicans* to form biofilms on the surfaces of medical and prosthetic devices, as well as on host epithelial and endothelial layers, is strongly related to a worse outcome [11]. *Candida* biofilms are notoriously resistant to antifungal therapy and severely compromise effective antifungal activity by phagocytes. Two papers in this Special Issue focus on specific antifungal immune mechanisms being impaired when confronted with *C. albicans* growing in biofilms. Kernien et al. demonstrate how various clinical *C. albicans* isolates forming biofilms inhibit the release of neutrophil extracellular traps (NETs) [12]. This inhibition was shown early in the formation of the biofilm before the development of long hyphal structures known to result in “frustrated” phagocytosis and release of NETs. Alonso et al. demonstrated an impaired migration of macrophages towards *C. albicans* growing in a biofilm compared to planktonic *Candida* cells [13]. Mannans in the fungal cell wall and/or secreted in the extracellular matrix seem to inhibit NET formation [14], while the impaired macrophage migration is not dependent on the presence of mannans. This is another example of how fungal polysaccharides play various and sometimes contradictory roles in the host–fungus interaction. 

Ongoing exploration and understanding of those immunological pathways is needed to ultimately lead to new directions in the development of immunotherapeutic strategies to spot the invading fungus early, leaving it no escape. Novel immunotherapeutic approaches are urgently needed, as the classes of antifungal drugs are restricted, and, even with the arrival of the newer ones, the prognosis for patients with fungal infection is still poor. A recent review by Armstrong-James and colleagues show the progress made in adjunctive antifungal strategies such as cytokine therapy, vaccines, and cellular immunotherapy [15]. 

I would like to take the opportunity to express my gratitude to our colleague experts who contributed to this Special Issue resulting in a collection of excellent papers summarizing current concepts and understanding as well as the latest insights obtained in the host–fungus interactions. 
